# The preparation of 3-substituted-1,5-dibromopentanes as precursors to heteracyclohexanes

**DOI:** 10.3762/bjoc.7.49

**Published:** 2011-03-31

**Authors:** Bryan Ringstrand, Martin Oltmanns, Jeffrey A Batt, Aleksandra Jankowiak, Richard P Denicola, Piotr Kaszynski

**Affiliations:** 1 Organic Materials Research Group, Department of Chemistry, Vanderbilt University, Box 1822 Station B, Nashville, TN 37235, USA, phone/fax: (615) 322-3458; 2 Faculty of Chemistry, University of Łódź, Tamka 12, 91403 Łódź, Poland

**Keywords:** 1,5-dibromopentanes, heterocycles, methodology, synthesis

## Abstract

The methodology to prepare 3-substituted 1,5-dibromopentanes **I** and their immediate precursors, which include 3-substituted 1,5-pentanediols **VII** or 4-substituted tetrahydropyrans **VIII**, is surveyed. Such dibromides **I** are important intermediates in the preparation of liquid crystalline derivatives containing 6-membered heterocyclic rings. Four dibromides **1a**–**1d** containing simple alkyl and more complex fragments at the 3-position were prepared. 3-Propyl- and 3-pentyl-pentane-1,5-diol (**2a**,**b**) were prepared starting from either glutaconate or malonate diesters, while tetrahydropyrans **3c** and **3d** were obtained from tetrahydro-4*H*-pyran-4-one. The advantages and disadvantages of each route are discussed. Dibromides **1c** and **1d** were used to prepare sulfonium zwitterions **11c** and **11d**.

## Introduction

**Figure 1:**
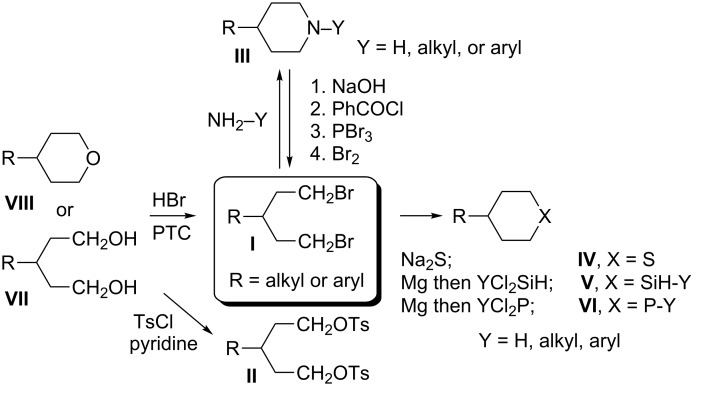
Methods for synthesis of dibromides **I** and their use for preparation of 6-membered heterocycles.

3-Substituted 1,5-dibromopentanes **I** and disulfonates, typically tosylates **II**, serve as useful intermediates in the preparation of six-membered heterocycles such as 4-substituted-piperidines **III**, thianes **IV**, silacyclohexanes **V**, and phosphorinanes **VI** (Figure 1). The piperidines **III** (Y = Ar) have been used as structural elements of liquid crystals [1–6] as well as antithrombotic and neuroprotectant agents [7–8]. Some thianes **IV** are found in petroleum distillates [9] and several have been used for the preparation of sulfones [7,10–11], sulfoxides [11–12], and sulfonium derivatives [13]. The latter, as well as silacyclohexanes **V** [14] and phosphorinanes **VI** [15–16], have been used in conformational and stereochemical studies.

The dibromides **I** or disulfonates **II** were prepared from 3-substituted 1,5-pentanediols **VII**. The former were obtained using HBr and concd sulfuric acid [2], while the latter from *p*-toluenesulfonyl chloride in the presence of a base such as pyridine [13,17–20]. Dibromides **I** can also be obtained by the ring-opening of tetrahydropyrans **VIII** under the same conditions employed for the diols **VII** [2,12,21–22], or via phase-transfer catalysis conditions with a phosphonium salt [23]. Piperidines **III** (Y = H) can undergo degradation to form dibromides **I** although this process is typically less efficient [12,14–15]. The piperidine ring can be reconstituted by ring-closure of **I** with an appropriate amine or aniline Y–NH_2_ [2,22]. The thianes **IV** are typically obtained from **I** or from **II** by reaction with Na_2_S [9–10,12–13], whereas the silacyclohexanes **V** and phosphorinanes **VI** are prepared by reacting the Grignard reagent derived from **I** with dichlorosilanes [14] or phosphonous dichlorides [15], respectively (Figure 1).

Our research program focuses on the development of polar, zwitterionic nematic liquid crystals having large longitudinal dipole moments for formulation of nematic materials with positive dielectric anisotropy [24–25]. The zwitterions consist of six-membered sulfonium rings attached to a boron cluster, either [*closo*-1-CB_9_H_10_]^−^ or [*closo*-1-CB_11_H_12_]^−^. Therefore, we have interest in cycloalkylating reagents such as dibromides **I** and disulfonates **II** containing alkyl or aryl substituents at the 3-position as intermediates for polar nematics. A number of such dibromides **I** and ditosylates **II** have been reported in the literature and some are listed in Table 1. For completeness, thianes **IV**, diols **VII**, and tetrahydropyrans **VIII** are also included.

**Table 1:** Selected compounds reported in the literature.

	R	Literature

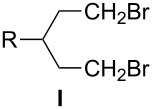	Me, *n*-Pr, *n*-Bu, C_5_H_11_, C_6_H_13_, C_7_H_15_, C_8_H_17_, C_9_H_19_, C_10_H_21_, Ph, 4-C_4_H_9_OPh, 4-ClPh	[2,9–10,12,14,21–22,26]
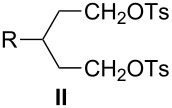	Me, Et, *n*-Pr, Ph, 4-CH_3_OPh, 4-C_5_H_11_Ph	[13,17–20]
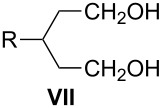	Me, Et, *n*-Pr, C_6_H_13_, Ph, 4-CH_3_OPh, 4-C_4_H_9_OPh, 4-ClPh, 4-C_5_H_11_Ph, PhCH_2_CH_2_	[2,9,17–20,26–28]
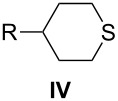	Me, Et, *n*-Pr, *n*-Bu, C_5_H_11_, C_6_H_13_, Ph, 4-ClPh, 4-HOPh, 4-NH_2_Ph	[7,9–13,29–30]
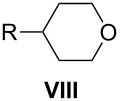	Me, *n*-Pr, *n*-Bu, C_5_H_11_, C_6_H_13_, C_7_H_15_, C_8_H_17_, C_9_H_19_, C_10_H_21_, C_11_H_23_, Ph, 4-HOPh, 4-C_4_H_9_OPh, 4-CF_3_Ph, 4-NH_2_Ph	[2,7,12,21–22,31–32]

**Scheme 1:**
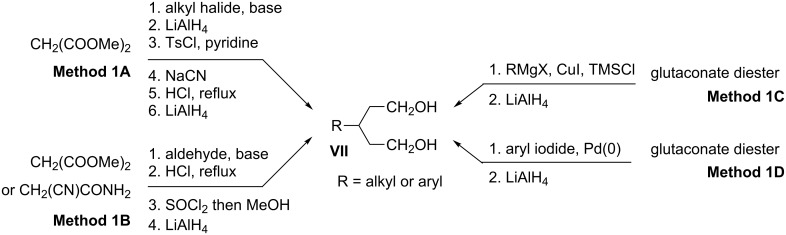
General methods for preparation of diols **VII**.

Diols **VII** represent a convenient intermediate for the preparation of dibromides **I** or ditosylates **II**, and four routes to **VII** are presented in Scheme 1. The first route (Method 1A) involves six steps starting from alkylation of malonate diester followed by reduction of the esters, tosylation of the alcohols, carbon homologation with NaCN, hydrolysis of the cyano groups to carboxylic acids, and lastly a second reduction to **VII**. Overall yields of diols **VII** using Method 1A are around 10% [27–28]. The second route (Method 1B) consists of a Knoevenagel condensation of malonate diester and an aldehyde followed by conjugate addition of a second equivalent of malonate diester. The tetraester is hydrolyzed, decarboxylated, esterified, and then reduced to give the diol. Yields for tetraesters are in the range of 25–85%, whereas the decarboxylation step gives the corresponding glutaric acids in 60–80% yield [19,33]. Variations of Method 1B involve the use of cyanoacetamide [34–36] or Meldrum’s acid [19,37] instead of malonate diester. For the former, the condensation with aldehyde is efficient and the product is crystalline, which simplifies its purification.

Another route to **VII** involves a two-step sequence of conjugate addition of an organocopper reagent to glutaconate diester [38] followed by reduction (Method 1C, Scheme 1). Yield of diols from this sequence are typically around 70% [17,38]. The downside to Method 1C is that an excess of Grignard reagent (3–4 equiv) is required; therefore, it is not economical with respect to the alkyl halide. Method 1D also uses glutaconate diester, which is reacted with an aryl iodide in the presence of Pd(0) under Heck conditions. The resulting unsaturated diester is reduced with excess LiAlH_4_ to give diol **VII** with an overall yield of about 30% for the two-step process [19].

**Scheme 2:**
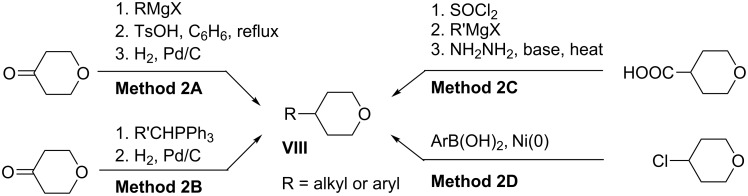
General methods for preparation of tetrahydropyrans **VIII**.

Tetrahydropyrans **VIII** represent a second general intermediate for access to dibromides **I**, and four routes are presented in Scheme 2. The first route involves the addition of a Grignard reagent to tetrahydro-4*H*-pyran-4-one, elimination of water, and hydrogenation of the olefin (Method 2A). Typical yields for Method 2A range from 20–30% [39–40]. The second route is the Wittig olefination of tetrahydro-4*H*-pyran-4-one followed by hydrogenation (Method 2B). Yields for the Wittig olefination of tetrahydro-4*H*-pyran-4-one range from 35–75% [41–43]. The third route (Method 2C) begins from tetrahydropyran-4-carboxylic acid: The acid chloride is reacted with a Grignard reagent, and the resulting ketone reduced under Wolff–Kischner conditions. Typical yields for this sequence are around 40% [21]. A recently described procedure [32] allows for efficient preparation of 4-aryltetrahydropyrans by the coupling of arylboronic acids and 4-chlorotetrahydropyran in the presence of Ni catalyst (Method 2D).

**Figure 2:**
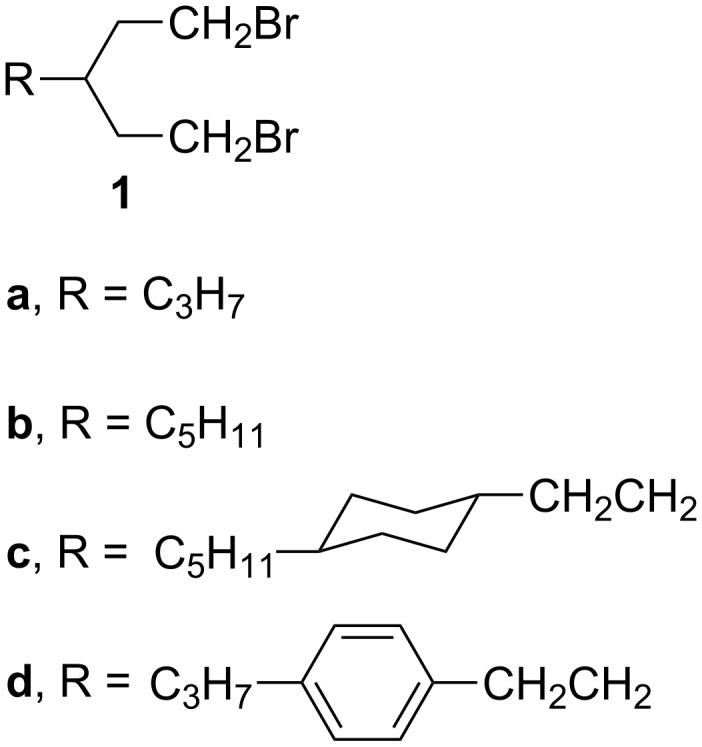
Structures of 1,5-dibromomopentanes **1a**–**1d**.

Here, we report the preparation of dibromides **1** having at the 3-position propyl (**1a**), pentyl (**1b**), 2-(4-*trans*-pentylcyclohexyl)ethyl (**1c**), or 4-propylphenethyl (**1d**) substituents (Figure 2). We investigate the preparation of appropriate diols **2** starting from malonate (Method 1B, Scheme 1) and glutaconate diesters (Method 1C) as well as appropriate tetrahydropyrans **3** via Wittig olefination (Method 2B, Scheme 2). These routes promise the minimal number of steps and ease of chemical transformations.

## Results

### Preparation of dibromides

**Scheme 3:**
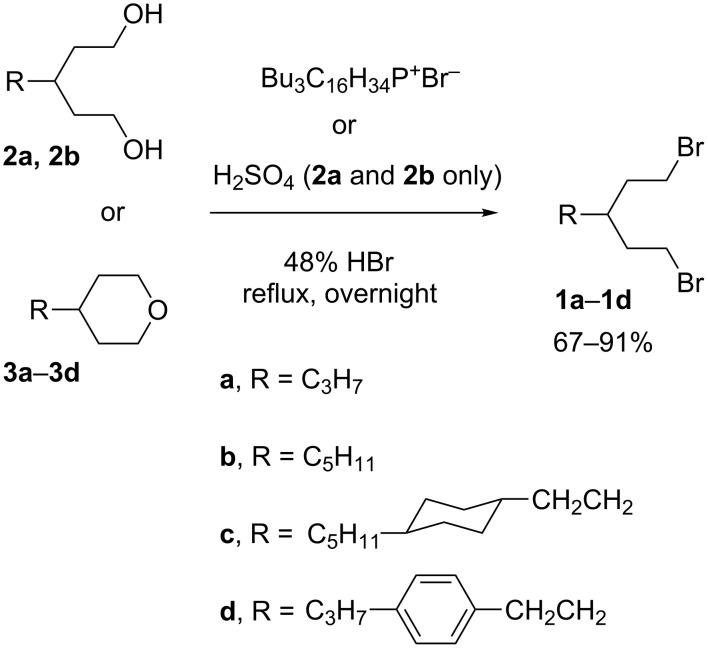
Preparation of dibromides **1**.

Dibromides **1a**–**1d** were prepared in 67–91% yield in refluxing 48% HBr with tributylhexadecylphosphonium bromide [44] as a phase-transfer catalyst either from diols **2a** and **2b** or tetrahydropyrans **3a**–**3d** (Scheme 3). When H_2_SO_4_ was used instead of the phosphonium salt, the yield of **1b** was lower (67%). The dibromides were purified on silica gel and, in addition, **1a** and **1b** were distilled.

### Preparation of diols

**Scheme 4:**
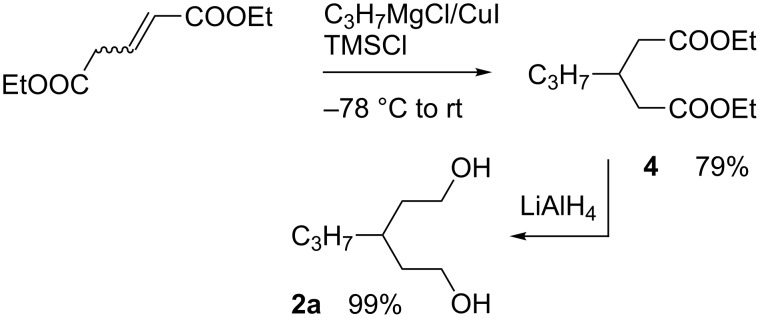
Preparation of diol **2a**.

Diols **2a** and **2b**, containing simple alkyl chains, were prepared using both the malonate and glutaconate routes, Method 1B and Method 1C, respectively (Scheme 1). The reaction of a 4-fold excess of propylmagnesium chloride with diethyl glutaconate in the presence of TMSCl with a catalytic amount of CuI generated diethyl 3-propylglutarate (**4**) in 79% yield. Reduction of **4** gave diol **2a** in nearly quantitative yield (Scheme 4).

**Scheme 5:**
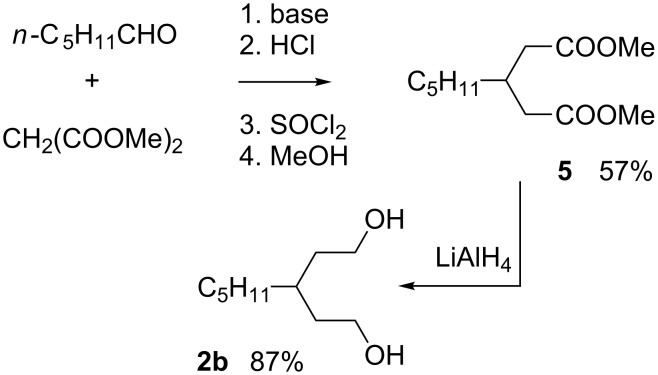
Preparation of diol **2b**.

Reaction of hexanal with excess dimethyl malonate under Knoevenagel conditions gave dimethyl 3-pentylglutarate (**5**) in 57% yield after decarboxylation and esterification (Scheme 5). 3-Propylglutarate **4** was obtained in a similar way from butanal in 49% overall yield. Diester **5** was subsequently reduced to give diol **2b** in 87% yield.

### Preparation of tetrahydropyrans

**Scheme 6:**
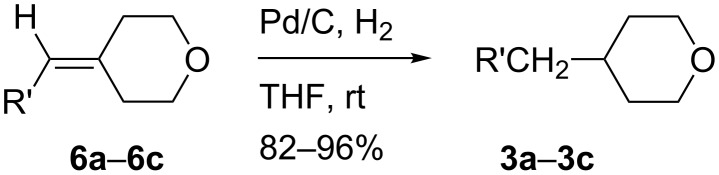
Preparation of tetrahydropyrans **3a**–**3c**.

Tetrahydropyrans **3a**–**3c** were prepared in 82%–96% yield by hydrogenation of the corresponding 4-methylenetetrahydropyrans **6a**–**6c** in THF (Scheme 6). Tetrahydropyrans **3a** and **3b** are volatile so care should be exercised during evaporation of solvent to ensure maximum yields.

**Scheme 7:**
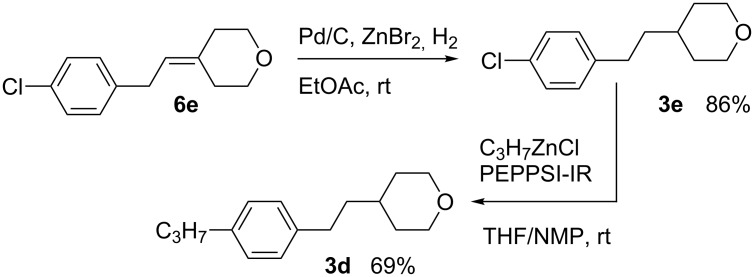
Preparation of tetrahydropyran **3d**.

Negishi coupling of tetrahydropyran **3e** with propylzinc chloride following a general literature method [45] using PEPPSI-IR as the Pd(0) source gave tetrahydropyran **3d** in 69% yield. Tetrahydropyran **3e** was prepared in 86% yield by hydrogenation of the corresponding 4-methylenetetrahydropyran **6e** in the presence of ZnBr_2_ to prevent reductive dechlorination (Scheme 7) [46]. Attempts to use diphenyl sulfide as a catalyst poison [47] resulted only in recovery of the starting material.

**Scheme 8:**
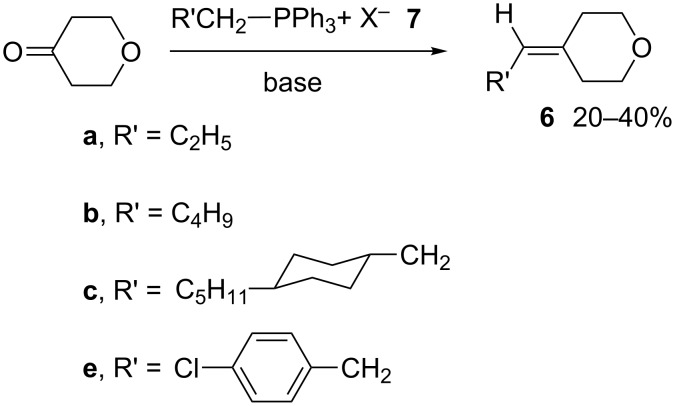
Preparation of methylenetetrahydropyrans **6**.

Wittig olefination of tetrahydro-4*H*-pyran-4-one gave substituted 4-methylenetetrahydropyrans **6a**–**6c** and **6e** in yields ranging from 20–40% (Scheme 8). Methylenetetrahydropyrans **6a** and **6b** are volatile compounds so again care should be taken during evaporation of solvent to maximize yields. Phosphoranes were generated using standard conditions by reacting phosphonium salts **7a**–**7c** and **7e** with either BuLi in anhydrous THF, or NaHMDS in a 1:1 mixture of anhydrous Et_2_O and anhydrous CH_2_Cl_2_.

**Scheme 9:**
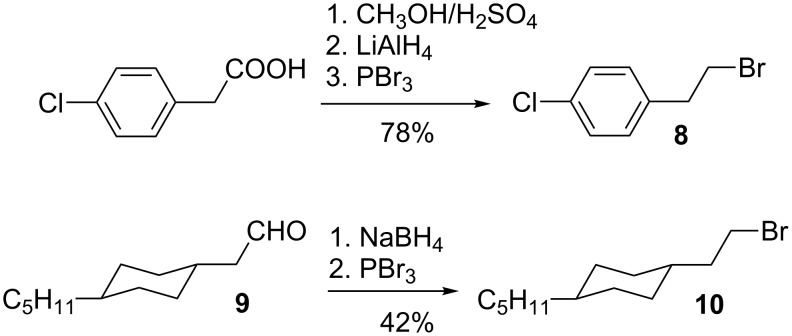
Preparation of bromides **8** and **10**.

Heating the corresponding alkyl halide (Br or I) with PPh_3_ under reflux in concentrated solutions of toluene, benzene, or acetonitrile gave phosphonium salts **7a**–7**c** and **7e**. Starting from 4-chlorophenylacetic acid, 4-chlorophenethyl bromide (**8**) was prepared in 78% overall yield according to the literature method [48] (Scheme 9). The phosphonium salt **7c**, an intermediate to dibromide **1c**, was prepared starting from the known 2-(*trans*-4-pentylcyclohexyl)acetaldehyde (**9**) [49]. The aldehyde was reduced with NaBH_4_ and subsequently brominated with PBr_3_ to give 1-bromo-2-(*trans*-4-pentylcyclohexyl)ethane (**10**) in overall yield of 42% (Scheme 9). Bromide **10** has been reported in the literature, but experimental details are lacking [50–51].

**Scheme 10:**
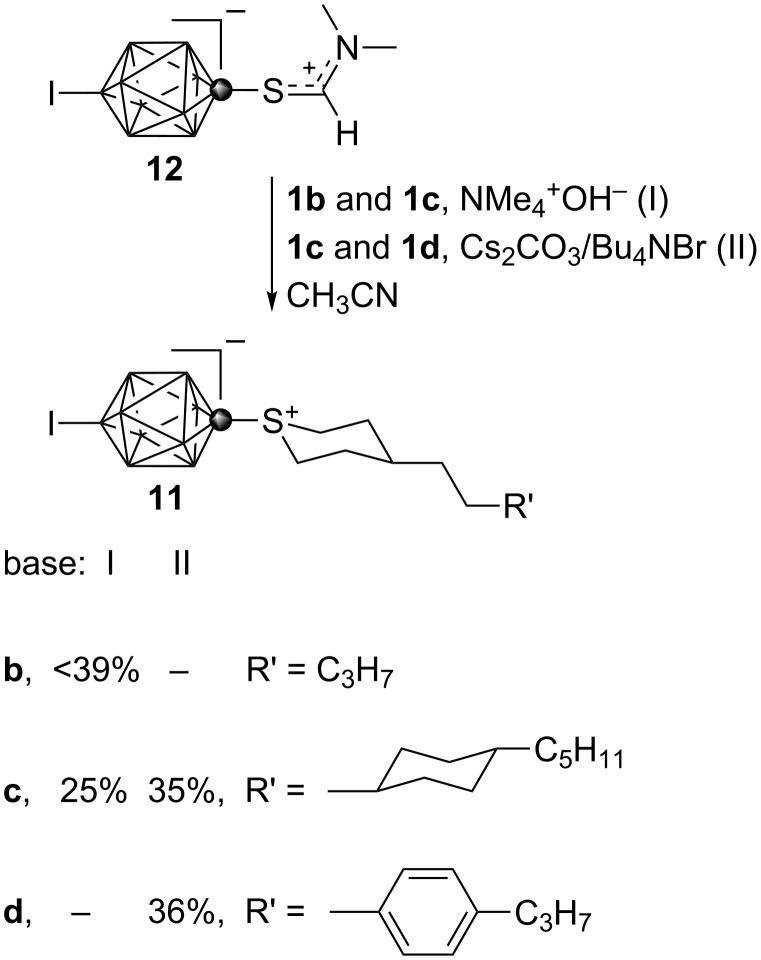
Preparation of sulfonium derivatives **11**.

The utility of dibromides **1** was demonstrated by preparation of sulfonium derivatives **11**. Thus, compound **11b** was previously obtained in 39% yield (before recrystallization) by alkylative cyclization of masked mercaptan [*closo*-1-CB_9_H_8_-1-SCHNMe_2_-10-I] **12** with dibromide **1b** in the presence of Me_4_N^+^OH^−^·5H_2_O in MeCN [25]. The same procedure gave **11c** in 25% yield after recrystallization, when starting from dibromide **1c**. Replacement of Me_4_N^+^OH^−^·5H_2_O with Cs_2_CO_3_ and a catalytic amount of Bu_4_N^+^Br^−^ in analogous reactions with dibromides **1c** and **1d** led to the sulfonium derivatives **11c** and **11d** in higher isolated yields of about 35% (Scheme 10). The higher yield for the cyclization in the presence of Cs_2_CO_3_ is attributed to less dehydrobromination of the electrophile than observed with Me_4_N^+^OH^−^.

## Discussion

Evaluation of the syntheses described here reveals advantages and disadvantages of the methods used for preparation of diols **VII** and tetrahydropyrans **VIII**. Both **VII** and **VIII** can be efficiently converted to dibromides **I** using HBr and tributylhexadecylphosphonium bromide as the phase-transfer catalyst.

Diols **VII** are obtained by reduction of a 3-substituted glutaric acid or ester. The latter can be prepared in a single step from glutaconate diester and a Grignard reagent. However, this method is practically limited to simple 3-alkyl-1,5-pentanediols, such as **1a** and **1b**, since large excess of Grignard reagents is required. The second method, in which glutaric acids are obtained from aldehydes and malonate ester, is more general and suitable for large scale synthesis, but involves an additional step.

Tetrahydropyrans **VIII** can be prepared in two steps via Wittig olefination. The efficiency of this method is limited by the initial olefination step of tetrahyro-4*H*-pyran-4-one, where the yields range from 20–40%. This step could potentially be improved by using different reaction conditions or an excess of the ylide. The hydrogenation of the corresponding olefins is straightforward and proceeds in high yield. In the case where a Pd-sensitive group is present, such as a chlorine substituent in **6e**, modification of the Pd catalyst with ZnBr_2_ permits efficient hydrogenation without loss of the halogen. The chlorophenyl derivative **3e** is a convenient precursor to a variety of 4-alkylphenyl and 4-arylphenyl derivatives that can be obtained via Negishi or Suzuki coupling methods (i.e., **3d**). Another such potentially general intermediate is 4-(4-hydroxyphenyl)tetrahydro-4*H*-pyran [31], which can be *O*-alkylated to provide 4-alkoxyphenyl derivatives [2].

Overall, we have demonstrated that dibromides **I** containing simple groups, such as propyl or pentyl, can be prepared in yields of about 70% starting from glutaconate diester (three steps, entry 2; Table 2), 36–38% from a malonate (four steps, entries 1 and 5), and on average 25% starting from tetrahydro-4*H*-pyran-4-one (three steps, entries 3 and 5). Dibromides **I** containing larger fragments such as 3-propylphenethyl or 2-(4-*trans*-pentylcyclohexyl)ethyl were prepared exclusively starting from tetrahydro-4*H*-pyran-4-one in overall yields averaging about 20% (three steps, entries 6 and 7). For comparison Table 2 includes the preparation of dibromides **I** (R = aryl) involving Heck-type arylation (entry 8) and dibromides **I** (R = alkyl) starting from tetrahydropyran-4-carboxylic acid (entry 9).

**Table 2:** Summary of dibromide **1** syntheses.

Entry	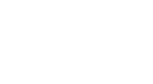	Route (Method)	Number of steps	Overall yield (%)

1	R = C_3_H_7_ (**1a**)	Malonate (1B)	4	36
2	R = C_3_H_7_ (**1a**)	Glutaconate (1C)	3	68
3	R = C_3_H_7_ (**1a**)	Pyranone (2B**)**	3	17
4	R = C_5_H_11_ (**1b**)	Malonate (1B)	4	38
5	R = C_5_H_11_ (**1b**)	Pyranone (2B)	3	33
6	R = C_5_H_11_C_6_H_10_CH_2_CH_2_ (**1c**)	Pyranone (2B)	3	20
7	R = C_3_H_7_C_6_H_4_CH_2_CH_2_ (**1d**)	Pyranone (2B)	4	18^a^
8	R = C_5_H_11_C_6_H_4_	Glutaconate (1D)	3	26^b^
9	R = C_3_H_7_–C_10_H_21_	4-COOH-pyran (2C)	4	40^c^

^a^4^th^ step includes replacement of Cl in Ar–Cl with propyl by Pd-catalyzed coupling reaction. Excluding this step the yield for the dibromide would have been 17% over three steps. ^b^Isolated as the ditosylate Ref. [19] ^c^Ref. [21].

It appears that the most economical way to prepare simple 3-alkyl-1,5-dibromopentanes is by using the aldehyde/malonate or aldehyde/cyanoacetamide method (Method 1B, Scheme 1), while the glutaconate method (Method 1C) is the simplest and most efficient. For the preparation of 3-aryl derivatives, the most direct methods are the Heck-type coupling with glutaconate ester (Method 1D) and Suzuki-type coupling to 4-chlorotetrahydropyran (Method 2D).

The preparation of dibromides **I** containing larger organic fragments from tetrahydropyrans **VIII** is inefficient and proceeds with low overall yields (Method 2B, Scheme 2). Attempts at conserving the alkylating reagent by using a stoichiometric Wittig olefination rather than conjugate addition of excess Grignard reagent to glutaconate did not give the expected result. It is possible that the aldehyde/cyanoacetamide method (Method 1B) [36] could prove advantageous and provide a synthesis which is more economical with respect to the alkyl substrate.

## Conclusion

Overall, several routes to dibromides **I** and ditosylates **II** were reviewed and investigated experimentally. These studies open the way for rational syntheses of other, specifically designed dielectrophiles that are important intermediates in the preparation of polar liquid crystals. In this context, we demonstrated the preparation of sulfonium derivatives **11c** and **11d** from dibromides **1c** and **1d** as precursors to such compounds. Other examples will be reported elsewhere.

## Supporting Information

File 1 General methods and synthetic procedures.

## References

[1] Adomenas P, Sirutkaitis R (1985). Mol Cryst Liq Cryst.

[2] Karamysheva L A, Kovshev E I, Pavluchenko A I, Roitman K V, Titov V V, Torgova S I, Grebenkin M F (1981). Mol Cryst Liq Cryst.

[3] Sheikh-Ali B M, Weiss R G (1991). Liq Cryst.

[4] Sheikh-Ali B M, Weiss R G (1994). Liq Cryst.

[5] Sucrow W, Schatull W (1982). Z Naturforsch, B.

[6] Tournilhac F, Nicoud J F, Simon J, Weber P, Guillon D, Skoulios A (1987). Liq Cryst.

[7] Pfefferkorn J A, Choi C, Winters T, Kennedy R, Chi L, Perrin L A, Lu G, Ping Y-W, McClanahan T, Schroeder R (2008). Bioorg Med Chem Lett.

[8] Mantegani S, Arlandini E, Brambilla E, Cremonesi P, Varasi M (2000). Synth Commun.

[9] Whitehead E V, Dean R A, Fidler F A (1951). J Am Chem Soc.

[10] Volynskii N P, Shcherbakova L P (1979). Bull Acad Sci USSR, Div Chem Sci (Engl Transl).

[11] Johnson C R (1963). J Am Chem Soc.

[12] Johnson C R, McCants D (1965). J Am Chem Soc.

[13] Halfpenny P J, Johnson P J, Robinson M J T, Ward M G (1976). Tetrahedron.

[14] Nguyen B T, Cartledge F K (1986). J Org Chem.

[15] Quin L D, Lee S O (1978). J Org Chem.

[16] Marsi K L, Jasperse J L, Llort F M, Kanne D B (1977). J Org Chem.

[17] Chang C-S, Lin Y-T, Shih S-R, Lee C-C, Lee Y-C, Tai C-L, Tseng S-N, Chern J-H (2005). J Med Chem.

[18] Cope A C, Cotter R J (1964). J Org Chem.

[19] Kaszynski P, Huang J, Jenkins G S, Bairamov K A, Lipiak D (1995). Mol Cryst Liq Cryst.

[20] Allinger N L, Neumann C L, Sugiyama H (1971). J Org Chem.

[21] Thomas J, Clough D (1963). J Pharm Pharmacol.

[22] Stapp P R, Drake C A (1971). J Org Chem.

[23] Bairamov K A, Douglass A G, Kaszynski P (1998). Synth Commun.

[24] Ringstrand B, Kaszynski P (2011). J Mater Chem.

[25] Ringstrand B, Kaszynski P, Januszko A, Young V G (2009). J Mater Chem.

[26] Allinger N L, Greenberg S (1959). J Am Chem Soc.

[27] Irwin A J, Lok K P, Huang K W-C, Jones J B (1978). J Chem Soc, Perkin Trans 1.

[28] Jones J B, Lok K P (1979). Can J Chem.

[29] Sarges R, Hank R F, Blake J F, Bordner J, Bussolotti D L, Hargrove D M, Treadway J L, Gibbs E M (1996). J Med Chem.

[30] Onesta R, Castelfranchi G (1959). Gazz Chim Ital.

[31] Koyama H, Boueres J K, Miller D J, Berger J P, MacNaul K L, Wang P-R, Ippolito M C, Wright S D, Agrawal A K, Moller D E (2005). Bioorg Med Chem Lett.

[32] González-Bobes F, Fu G C (2006). J Am Chem Soc.

[33] Boots M R, Yeh Y-M, Boots S G (1980). J Pharm Sci.

[34] Farmer E H, Martin S R W (1933). J Chem Soc.

[35] Curtis R H, Day J N E, Kimmins L G (1923). J Chem Soc, Trans.

[36] Day J N E, Thorpe J F (1920). J Chem Soc, Trans.

[37] Hedge J A, Kruse C W, Snyder H R (1961). J Org Chem.

[38] Leotta G J, Overman L E, Welmaker G S (1994). J Org Chem.

[39] Booth H, Khedhair K A, Readshaw S A (1987). Tetrahedron.

[40] Chini M, Crotti P, Gardelli C, Macchia F (1994). Tetrahedron.

[41] Margot C, Rizzolio M, Schlosser M (1990). Tetrahedron.

[42] Kabat M M, Lange M, Wovkulich P M, Usokovic M R (1992). Tetrahedron Lett.

[43] Angelastro M R, Baugh L E, Bey P, Burkhart J P, Chen T-M, Durham S L, Hare C M, Huber E W, Janusz M J, Koehl J R (1994). J Med Chem.

[44] Landini D, Montanari F, Rolla F (1978). Synthesis.

[45] Organ M G, Avola S, Dubovyk I, Hadei N, Kantchev E A B, O'Brien C J, Valente C (2006). Chem–Eur J.

[46] Wu G, Huang M, Richards M, Poirier M, Wen X, Draper R W (2003). Synthesis.

[47] Mori A, Mizusaki T, Kawase M, Maegawa T, Monguchi Y, Takao S, Takagi Y, Sajiki H (2008). Adv Synth Catal.

[48] Lambert J B, Mark H W, Magyar E S (1977). J Am Chem Soc.

[49] Jankowiak A, Januszko A, Ringstrand B, Kaszynski P (2008). Liq Cryst.

[50] Dąbrowski R, Dziaduszek J, Szczuciński T, Parka J (1995). Mol Cryst Liq Cryst.

[51] Fearon J E, Gray G W, Ifill A D, Toyne K J (1985). Mol Cryst Liq Cryst.

