# Supplementary material for: The preparation of 3-substituted-1,5-dibromopentanes as precursors to heteracyclohexanes
Source: Beilstein J Org Chem. 2011 Mar 31;7:386–93. doi: 10.3762/bjoc.7.49 (PMC3079112; doi:10.3762/bjoc.7.49)
Supplement: File 1 — General methods and synthetic procedures. [file Beilstein_J_Org_Chem-07-386-s001.pdf]

Supporting Information

for

The preparation of 3-substituted-1,5-dibromopentanes as precursors to heteracyclohexanes

Bryan Ringstrand[1], Martin Oltmanns[1,§], Jeffrey A. Batt[1,§], Aleksandra Jankowiak[1], Richard P. Denicola[1] and Piotr Kaszynski[*1,2]

Address: [1]Organic Materials Research Group, Department of Chemistry, Vanderbilt University, Box 1822 Station B, Nashville, TN 37235, USA, Phone/fax: (615) 322-3458 and [2]Faculty of Chemistry, University of Łódź, Tamka 12, 91403 Łódź, Poland

Email: Piotr Kaszynski - piotr.kaszynski@vanderbilt.edu

[*]Corresponding author

[§]Summer research students visiting from Augustana College, Rock Island, IL.

General methods and synthetic procedures

Table of Context

1. Experimental …S2

2. References …S20

1. Experimental

Reagents and solvents were obtained commercially. THF was distilled from potassium containing benzophenone, and Et_2O was distilled from sodium containing benzophenone. NMP was dried over 4 Å MS, and TMSCl was distilled from CaH_2. Butyllithium was titrated against menthol with 1,10-phenanthroline as indicator. Grignard reagents were titrated against molecular iodine in 0.5 M LiCl solution in anhydrous THF [1]. Reactions were carried out under an Ar atmosphere, and all subsequent manipulations were conducted in air. NMR spectra were obtained at 400.1 MHz (^{1}H), 100 MHz (^{13}C), and 162 MHz (^{31}P) in $CDCl_3$, unless otherwise specified. ^{1}H NMR and ^{13}C NMR spectra were referenced to the residual solvent peaks. Melting points and boiling points are uncorrected.

1,5-Dibromopentanes 1. A general procedure.

Method A. Following a general literature procedure [2], a biphasic mixture of the 4-substituted tetrahydropyran **3** or diol **2**, 47% aqueous HBr (15 equiv) and $Bu_3C_{16}H_{33}P^+Br^-$ (0.1 equiv) was stirred at 100 °C overnight under an Ar atmosphere. The black reaction mixture was cooled to rt, diluted by the addition of half its volume of H_2O, and extracted with CH_2Cl_2 or petroleum ether (3×). The organic layers were combined, dried ($MgSO_4$) and evaporated to leave a black residue. The residue was passed through a short plug of silica gel (hexanes as eluent) to give the dibromide as slightly yellow oil. The dibromide was further purified by distillation in the case of **1a** and **1b**. Due to the anticipated high boiling point of **1c** and **1d**, the compounds were purified only on silica gel.

1,5-Dibromo-3-propylpentane (1a) [3].

Prepared in 71% yield from 4-propyltetrahydropyran (**3a**, 1.70 g) or in 75% yield from 3-propyl-1,5-pentanediol (**2a**, 21.0 g). A colorless oil: bp 160–170 °C (13 mmHg) Kugelrohr or 75–79 (0.3 mmHg); Lit.[3] bp 147 °C (17 mmHg); ^{1}H NMR (400 MHz, $CDCl_3$) δ 0.91 (t, *J* = 6.9 Hz, 3H), 1.22–1.33 (m, 4H), 1.72–1.77 (m, 1H), 1.78–1.92 (m, 4H), 3.42 (t, *J* = 7.3 Hz, 4H); ^{13}C NMR (100 MHz, $CDCl_3$) δ 14.3, 19.3, 31.2 (2C), 34.8, 35.3, 36.7 (2C). Anal. Calcd. for $C_8H_{16}Br_2$: C, 35.32; H, 5.93. Found: C, 35.56; H, 6.02.

1,5-Dibromo-3-pentylpentane (1b) [3].

Prepared in 87% yield from 4-pentyltetrahydropyran (**3b**, 1.20 g) or in 67% yield from 3-pentyl-1,5-pentanediol (**2b**, 20.7 g) with an equal volume of conc. H_2SO_4 (relative to HBr) instead of $Bu_3C_{16}H_{33}P^+Br^-$. Colorless oil: bp 82–83 °C (0.15 mmHg); Lit.[3] bp 157 °C (13 mmHg); ^{1}H NMR (400 MHz, $CDCl_3$) δ 0.91 (t, *J* = 6.9 Hz, 3H), 1.23–1.34 (m, 8H), 1.70–1.77 (m, 1H), 1.78–1.92 (m, 4H), 3.42 (t, *J* = 7.3 Hz, 4H); ^{13}C NMR (100 MHz, $CDCl_3$) δ 14.1, 22.6, 25.8, 31.3 (2C), 32.1, 32.5, 35.5, 36.7 (2C); EIMS, *m*/*z* 302, 300, and 298 (1:2:1, M+, 1), 219 and 221(1:1 M−Br, 6), 69 (100). Anal. Calcd. for $C_{10}H_{20}Br_2$: C, 40.03; H, 6.72. Found: C, 40.50; H, 6.76.

1,5-Dibromo-3-(2-(*trans*-4'-pentylcyclohexyl)ethyl)pentane (1c).

Prepared in 91% yield from 4-(2-(*trans*-4'-pentylcyclohexyl)ethyl)tetrahydropyran (**3c**, 0.90 g) and obtained as a slight yellow oil: ^{1}H NMR (400 MHz, $CDCl_3$) δ 0.88 (t, *J* = 7.0 Hz, 3H), 0.80–0.92 (m, 4H), 1.09–1.19 (m, 5H), 1.20–1.34 (m, 10H), 1.67–1.76 (m, 4H), 1.77–1.91 (m, 4H), 3.41 (t, *J* = 7.3 Hz, 4H); ^{13}C NMR (100 MHz, $CDCl_3$) δ 14.1, 22.7,

26.6, 29.7, 31.3 (2C), 32.2, 33.2 (2C), 33.3 (2C), 33.6, 35.7, 36.6 (2C), 37.4, 37.8, 38.1; TOF MS (EI+): calcd for $C_{18}H_{34}Br_2$: *m/z* = 408.1027; found: 408.1029. Anal. Calcd. for $C_{18}H_{34}Br_2$: C, 52.70; H, 8.35. Found: C, 53.60; H, 8.48.

1,5-Dibromo-3-(2-(4-propylphenyl)ethyl)pentane (1d).

Prepared in 76% yield from 4-(2-(4-propylphenyl)ethyl)tetrahydropyran (**3d**, 2.30 g) and obtained as a slight yellow oil: ^{1}H NMR (400 MHz, $CDCl_3$) δ 0.94 (t, *J* = 7.3 Hz, 3H), 1.57–1.68 (m, 4H), 1.79–1.86 (m, 1H), 1.88–1.98 (m, 4H), 2.53–2.61 (m, 4H), 3.43 (t, *J* = 7.2 Hz, 4H), 6.99–7.12 (m, 4H); ^{13}C NMR (100 MHz, $CDCl_3$) δ 13.9, 24.6, 30.9 (2C), 32.2, 34.6, 35.4, 36.6 (2C), 37.6, 128.1 (2C), 128.5 (2C), 139.0, 140.3; TOF MS (EI+): calcd for $C_{16}H_{24}Br_2$: *m/z* = 374.0245; found: 374.0232. Anal. Calcd. for $C_{16}H_{24}Br_2$: C, 51.09; H, 6.43. Found: C, 52.30; H, 6.80.

Preparation of 3-propylpentane-1,5-diol (2a) [4].

A solution of diethyl 3-propylglutarate (**4**, 4.9 g, 21.3 mmol) in anhydrous THF (50 mL) was added dropwise to a suspension of $LiAlH_4$ (1.62 g, 42.6 mmol) in anhydrous THF (150 mL) at 0 °C in a 500 mL 3 necked flask under an Ar atmosphere. The reaction was warmed to rt and gently heated under reflux overnight. The reaction was cooled to 0 °C, carefully quenched with H_2O (10 mL) and then 2 M KOH (25 mL). After 30 minutes, the white precipitate was removed by filtration and washed with Et_2O (100 mL). The filtrate was dried ($MgSO_4$), filtered and evaporated to give 3.10 g (99% yield) of 3-propyl-1,5-pentanediol (**2a**) as a colorless oil which was used without further purification: ^{1}H NMR (500 MHz, $CDCl_3$) δ 0.90 (t, *J* = 6.8 Hz, 3H), 1.24–1.36 (m, 4H), 1.49–1.67 (m, 5H),

1.79 (s, 2H), 3.64–3.76 (m, 4H); ^{13}C NMR (100 MHz, $CDCl_3$) δ 14.4, 19.7, 31.1, 36.6 (2C), 36.9, 61.0 (2C).

Preparation of 3-pentylpentane-1,5-diol (2b).

Dimethyl 3-pentylglutarate (**5**, 31.3 g, 0.136 mol) was reduced with $LiAlH_4$ (7.60 g, 0.20 mol) in dry Et_2O (500 mL) as described for **2a**. The reaction mixture was decomposed by the successive addition of Et_2O saturated with H_2O (100 mL), H_2O (8 mL), 15% NaOH (8 mL) and H_2O (25 mL). The resulting suspension was filtered through Celite, evaporated and dried in vacuo. The resulting crude oily diol (20.7 g, 87% yield) was used for the preparation of **1b** without further purification. ^{1}H NMR (400 MHz, $CDCl_3$) δ 0.88 (t, J = 6.9 Hz, 3H), 1.24–1.36 (m, 8H), 1.52–1.68 (m, 7H), 3.65–3.76 (m, 4H).

Preparation of tetrahydropyrans 3. General procedure for the hydrogenation of 4-methylenetetrahydropyrans 6.

Procedure A. A suspension of anhydrous THF (15 mL), olefin **6** (25 mmol), 10% Pd/C (10 wt %) was hydrogenated by the balloon method (760 mmHg). The reaction progress was monitored by ^{1}H NMR until starting material was no longer observed. The suspension was filtered through Celite and evaporated. The pyran was further purified by passage through a short plug of silica gel (eluent: CH_2Cl_2/hexane, 1:9) and by bulb-to-bulb distillation.

Procedure B. Following a modified literature procedure [5], a suspension of EtOAc (15 mL), olefin **6** (25 mmol), 10% Pd/C (10 wt %) and anhydrous $ZnBr_2$ (0.2 equiv) was

hydrogenated by the balloon method. The reaction was monitored and products isolated as in Procedure A.

4-Propyltetrahydro-2*H*-pyran (3a) [3].

Prepared from 1.70 g of **6a** according to Procedure A in 82% yield and obtained as a colorless oil: bp 100–110 °C (55 mmHg); Lit.[3] bp 55–56 °C (11 mmHg) ^{1}H NMR (400 MHz, $CDCl_3$) δ 0.89 (t, J = 7.2 Hz, 3H), 1.18–1.37 (m, 6H), 1.41–1.52 (m, 1H), 1.54–1.62 (m, 2H), 3.36 (td, J_1 = 11.7 Hz, J_2 = 2.0 Hz, 2H), 3.94 (ddd, J_1 = 10.9 Hz, J_2 = 4.1 Hz, J_3 = 1.2 Hz, 2H); ^{13}C NMR (100 MHz, $CDCl_3$) δ 14.2, 19.4, 33.2 (2C), 34.7, 39.2, 68.2 (2C). Anal. Calcd. for $C_8H_{16}O$: C, 74.94; H, 12.58. Found: C, 74.66; H, 12.81.

4-Pentyltetrahydro-2*H*-pyran (3b) [3].

Prepared from 1.30 g of **6b** according to Procedure A in 94% yield and obtained as a colorless oil: bp 155–160 °C (55 mmHg); Lit.[3] bp 83–84 °C (15 mmHg); ^{1}H NMR (500 MHz, $CDCl_3$) δ 0.88 (t, J = 7.0 Hz, 3H), 1.18–1.34 (m, 10H), 1.40–1.50 (m, 1H), 1.58 (br d, J = 13.0 Hz, 2H), 3.36 (td, J_1 = 11.7 Hz, J_2 = 1.8 Hz, 2H), 3.94 (dd, J_1 = 11.1 Hz, J_2 = 4.3 Hz, 2H); ^{13}C NMR (100 MHz, $CDCl_3$) δ 14.2, 22.8, 26.2, 32.2, 33.4 (2C), 35.1, 37.1, 68.4 (2C). Anal. Calcd. for $C_{10}H_{20}O$: C, 76.86; H, 12.90. Found: C, 76.23; H, 12.93.

4-(2-(*trans*-4-Pentylcyclohexyl)ethyl)tetrahydro-2*H*-pyran (3c).

Prepared from 0.90 g of **6c** according to Procedure A in 96% yield and obtained as a colorless oil: bp 235–240 °C (0.5 mmHg); ^{1}H NMR (400 MHz, $CDCl_3$) δ 0.79–0.92 (m, 4H), 0.88 (t, J = 7.0 Hz, 3H), 1.05–1.33 (m, 16H), 1.34–1.46 (m, 1H), 1.54–1.62 (m, 2H),

1.72 (d, J = 8.5 Hz, 4H), 3.36 (td, J_1 = 11.7 Hz, J_2 = 1.9 Hz, 2H), 3.94 (ddd, J_1 = 11.2 Hz, J_2 = 3.7 Hz, J_3 = 1.0 Hz, 2H); ^{13}C NMR (100 MHz, $CDCl_3$) δ 14.1, 22.7, 26.7, 32.2, 33.30 (2C), 33.35 (2C), 33.40 (2C), 34.1, 34.3, 35.3, 37.5, 37.9, 38.0, 68.2 (2C). Anal. Calcd. for $C_{18}H_{34}O$: C, 81.13; H, 12.86. Found: C, 81.34; H, 12.83.

4-(4-Chlorophenethyl)tetrahydro-2*H*-pyran (3e).

Prepared from 3.90 g of **6e** according to Procedure B in 86% yield and obtained as a colorless oil: bp 180–185 °C (0.2 mmHg); ^{1}H NMR (400 MHz, $CDCl_3$) δ 1.25–1.37 (m, 2H), 1.43–1.58 (m, 3H), 1.63 (br d, J = 13.2 Hz, 2H), 2.60 (t, J = 7.8 Hz, 2H), 3.36 (td, J_1 = 11.8 Hz, J_2 = 2.0 Hz, 2H), 3.96 (ddd, J_1 = 11.0 Hz, J_2 = 4.0 Hz, J_3 = 1.1 Hz, 2H), 7.10 (d, J = 8.5 Hz, 2H), 7.24 (d, J = 8.4 Hz, 2H); ^{13}C NMR (100 MHz, $CDCl_3$) δ 32.0, 33.0 (2C), 34.4, 38.6, 68.0 (2C), 128.4 (2C), 129.6 (2C), 131.4, 140.9. Anal. Calcd. for $C_{13}H_{17}ClO$: C, 69.48; H, 7.62. Found: C, 69.71; H, 7.68.

Preparation of 4-(4-propylphenethyl)tetrahydro-2*H*-pyran (3d).

Following a modified literature procedure for the coupling of aryl chlorides to alkylzinc reagents [6], a solution of 4-(4-chlorophenethyl)tetrahydro-2*H*-pyran (**3e**, 3.17 g, 14.1 mmol) in anhydrous THF (5 mL) was added to a homogeneous solution of propylmagnesium chloride (21 mL, 42 mmol, 2 M in Et_2O), anhydrous $ZnCl_2$ (6.75 g, 49.5 mmol), anhydrous LiCl (3.59 g, 85 mmol), and PEPPSI-IR (0.202 g, 0.3 mmol, 2 mol %) in a 2:1 mixture of anhydrous NMP/THF (150 mL). The reaction became exothermic, the solution became dark brown and was left to stir overnight at rt. Reaction progress was monitored by ^{1}H NMR and additional catalyst or organozinc reagent was

added if necessary. The reaction was quenched with CH_3OH, the reaction mixture filtered, and excess solvent removed in vacuo. 10% HCl (100 mL) was added to the dark brown viscous oil, and the suspension extracted with hexanes (4 × 30 mL). The organic layers were combined, dried ($MgSO_4$) and evaporated to give 3.81 g of a yellow oil. The yellow oil was passed through a short plug of silica gel (eluent: CH_2Cl_2/hexane, 1:9) to afford 2.45 g of a colorless oil which was further purified by bulb-to-bulb distillation to furnish 2.25 g (69% yield) of 4-(4-propylphenethyl)tetrahydro-2*H*-pyran (**3d**): bp 165–170 °C (0.2 mmHg); ^{1}H NMR (400 MHz, $CDCl_3$) δ 0.94 (t, J = 7.3 Hz, 3H), 1.25–1.38 (m, 2H), 1.46–1.69 (m, 7H), 2.56 (t, J = 7.7 Hz, 2H), 2.60 (t, J = 7.9 Hz, 2H), 3.37 (td, J_1 = 11.7 Hz, J_2 = 2.0 Hz, 2H), 3.96 (ddd, J_1 = 10.9 Hz, J_2 = 4.0 Hz, J_3 = 1.0 Hz, 2H), 7.07–7.12 (m, 4H); ^{13}C NMR (100 MHz, $CDCl_3$) δ 13.9, 24.6, 32.2, 33.1 (2C), 34.5, 37.6, 38.8, 68.1 (2C), 128.1 (2C), 128.4 (2C), 139.7, 140.0. Anal. Calcd. for $C_{16}H_{24}O$: C, 82.70; H, 10.41. Found: C, 82.98; H, 10.26.

Preparation of diethyl 3-propylglutarate (4) [7].

Method A. Following a modified literature protocol [8], a solution of propylmagnesium chloride (54 mL, 107.6 mmol, 2.0 M in Et_2O) was added to a suspension of CuI (1.5 g, 8.07 mmol) in anhydrous THF (200 mL) in a 500 mL, 3-necked flask under a stream of N_2. The resulting black solution was cooled to −78 °C, and freshly distilled TMSCl (17 mL, 134.5 mmol) added slowly followed by diethyl glutaconate (5.0 g, 26.9 mmol). The solution was stirred at −78 °C for 2 h and then stored in the freezer at −15 °C overnight under an Ar atmosphere. The reaction was then warmed to rt and carefully quenched with saturated aqueous NH_4Cl (100 mL). The biphasic mixture was filtered, the organic layer

separated, and the aqueous layer further extracted with Et_2O (3 × 25 mL). The organic layers were combined, dried ($MgSO_4$) and evaporated to give 6.8 g of a red oil. The crude product was passed through a short plug of silica gel (eluent: CH_2Cl_2/hexane, 1:1, R_f = 0.2) to afford 5.3 g of a slightly yellow oil. The oil was further purified by distillation (95–97 °C, 0.5 mmHg; lit.[7] bp 132 °C, 10 mmHg) to give 4.9 g (79% yield) of diethyl 3-propylglutarate (**4**) as a colorless oil: ^{1}H NMR (400 MHz, $CDCl_3$) δ 0.86–0.92 (m, 3H), 1.25 (t, J = 7.1 Hz, 6H), 1.30–1.35 (m, 4H), 2.34 (br s, 5H), 4.12 (q, J = 7.1 Hz, 4H); ^{13}C NMR (100 MHz, $CDCl_3$) δ 14.1, 14.2 (2C), 19.7, 31.9, 36.2, 38.6 (2C), 60.3 (2C), 172.6 (2C). Anal. Calcd. for $C_{12}H_{22}O_4$: C, 62.58; H, 9.63. Found: C, 65.84; H, 9.80.

Method B. Following the method described for the preparation of **5** and substituting EtOH for MeOH, diethyl 3-propylglutarate **4** was obtained in 49% yield (33.5 g).

Preparation of dimethyl 3-pentylglutarate (5).

A mixture of dimethyl malonate (85.8 g, 0.65 mol), freshly distilled hexanal (30.0 g, 0.30 mol), benzene (50 mL), piperidine (1 mL), and NEt_3 (3 mL) was stirred at rt for 2 h and then heated overnight under reflux. A Dean-Stark trap was used to collect water produced in the reaction. The mixture was washed with dilute HCl, dried, and excess dimethyl malonate was removed under vacuum (up to 80 °C, 0.4 mmHg). The oily residue was heated overnight under reflux with conc. HCl (250 mL), the aqueous acid was removed under reduced pressure, and the oily residue was subjected to short path-distillation (150–160 °C, 0.5 mmHg) to give crude oily diacid (41.8 g). Without further purification, the diacid was heated under reflux with $SOCl_2$ (60 mL) for 2 h, the excess $SOCl_2$ was removed under reduced pressure, and the resulting dark acid chloride was heated under

reflux with CH_3OH (200 mL) for 2 h. Excess CH_3OH was evaporated, and crude diester distilled under high vacuum (100–101 °C, 0.4 mmHg) to give 39.33 g (57% overall yield) of dimethyl 3-pentylglutarate (**5**) as a colorless oil: ^{1}H NMR (300 MHz, $CDCl_3$) δ 0.88 (t, *J* = 6.5 Hz, 3H), 1.20–1.35 (m, 8H), 2.35 (s, 5H), 3.67 (s, 6H); ^{13}C NMR (75 MHz, $CDCl_3$) δ 13.9, 22.5, 26.2, 31.7, 32.1, 33.9, 38.3, 51.4, 173.0; IR, (C=O) 1737 cm^{-1}; EIMS, *m*/*z* 199 (M–MeO, 59), 125 (100). Anal. Calcd for $C_{12}H_{22}O_4$: C, 62.58; H, 9.63. Found: C, 62.35; H, 9.72.

Preparation substituted 4-methylenetetrahydropyrans 6. A general procedure.

Procedure A. A suspension of phosphonium salt **7** (50 mmol) in anhydrous THF (150 mL) was treated with butyllithium (50 mmol) at 0 °C and stirred for 1 h under an Ar atmosphere. A solution of tetrahydropyran-4-one (50 mmol) in anhydrous THF (50 mL) was added dropwise to the deep red/orange solution of the phosphorane . The solution was brought to rt and stirred overnight. Saturated NH_4Cl (200 mL) was added to discharge the red color, the mixture diluted with Et_2O, and the organic layer separated. The aqueous layer was further extracted with Et_2O (3 × 50 mL). The combined organic layers were dried ($MgSO_4$), filtered and evaporated. The product was passed through a short plug of silica gel (eluent: CH_2Cl_2/hexane, 1:9) and further purified by bulb-to-bulb distillation to give the pure olefin.

Procedure B. Following a modified literature procedure [9], a solution of the phosphonium salt **7** (70 mmol) in anhydrous CH_2Cl_2 (200 mL) and anhydrous Et_2O (200 mL) at 0 °C was treated with sodium bis(trimethysilyl)amide (1.0 M in THF, 75 mL, 75 mmol) under an Ar atmosphere. The deep red solution of phosphorane was maintained at

0 °C for 1 h, and a solution of tetrahydropyran-4-one (50 mmol, 0.7 equiv) in anhydrous CH_2Cl_2 (20 mL) added dropwise. The reaction mixture was stirred overnight at rt and diluted with sat. NH_4Cl (100 mL) to yield a red biphasic solution. The excess organic solvents were removed in vacuo, and the aqueous layer treated with hexane (200 mL) to yield an orange precipitate. The mixture was filtered and washed with additional hexane. The hexane layer was separated, and the aqueous layer further extracted with hexane (3 × 50 mL). The combined organic layers were dried ($MgSO_4$), filtered and evaporated. The product was passed through a short plug of silica gel (eluent: CH_2Cl_2/hexane, 1:9) and further purified by bulb-to-bulb distillation to giving the pure olefin.

4-(Propylidene)tetrahydro-2*H*-pyran (6a).

Prepared from 21.6 g of **7a** according to **Procedure A** in 30% yield and obtained as a colorless oil: bp 100–110 °C (55 mmHg); ^{1}H NMR (500 MHz, $CDCl_3$) δ 0.95 (t, *J* = 7.5 Hz, 3H), 2.00 (quint, *J* = 7.4 Hz, 2H), 2.19 (t, *J* = 5.2 Hz, 2H), 2.26 (t, *J* = 5.3 Hz, 2H), 3.66 (quint, *J* = 5.5 Hz, 4H), 5.19 (t, *J* = 7.2 Hz, 1H); ^{13}C NMR (100 MHz, $CDCl_3$) δ 14.8, 20.3, 29.8, 37.0, 69.0, 69.9, 125.3, 133.6. Anal. Calcd. for $C_8H_{14}O$: C, 76.14; H, 11.18. Found: C, 76.05; H, 11.42.

4-(Pentylidene)tetrahydro-2*H*-pyran (6b).

Prepared from 9.20 g of **7b** according to **Procedure A** in 40% yield and obtained as a colorless oil: bp 155–160 °C (55 mmHg); ^{1}H NMR (400 MHz, $CDCl_3$) δ 0.89 (t, *J* = 7.1 Hz, 3H), 1.25–1.37 (m, 4H), 1.99 (q, *J* = 7.1 Hz, 2H), 2.19 (t, *J* = 5.1 Hz, 2H), 2.25 (t, *J* = 5.1 Hz, 2H), 3.64 (t, *J* = 5.5 Hz, 2H), 3.66 (t, *J* = 5.5 Hz, 2H), 5.18 (t, *J* = 7.3 Hz, 1H);

^{13}C NMR (100 MHz, $CDCl_3$) δ 14.0, 22.3, 26.6, 29.7, 32.2, 37.0, 68.9, 69.7, 123.5, 133.9. Anal. Calcd. for $C_{10}H_{18}O$: C, 77.87; H, 11.76. Found: C, 77.59; H, 11.78.

4-(2-(*trans*-4'-Pentylcyclohexyl)ethylidene)tetrahydro-2*H*-pyran (6c).

Prepared from 7.90 g of **7c** according to **Procedure A** in 23% yield and obtained as a colorless oil: bp 230 °C (0.2 mmHg); ^{1}H NMR (400 MHz, $CDCl_3$) δ 0.79–0.94 (m, 4H), 0.88 (t, *J* = 7.0 Hz, 3H), 1.10–1.34 (m, 10H), 1.72 (d, *J* = 8.8 Hz, 4H), 1.88 (t, *J* = 7.1 Hz, 2H), 2.20 (t, *J* = 5.2 Hz, 2H), 2.25 (t, *J* = 5.1 Hz, 2H) 3.64 (t, *J* = 5.5 Hz, 2H), 3.67 (t, *J* = 5.5 Hz, 2H), 5.20 (t, *J* = 7.5 Hz, 1H); ^{13}C NMR (100 MHz, $CDCl_3$) δ 14.1, 22.7, 26.7, 29.8, 32.2, 33.1 (2C), 33.3 (2C), 34.7, 37.1, 37.4, 37.8, 38.7, 68.8, 69.8, 122.1, 134.5. Anal. Calcd. for $C_{18}H_{32}O$: C, 81.75; H, 12.20. Found: C, 81.72; H, 12.24.

4-(2-(4-Chlorophenyl)ethylidene)tetrahydro-2*H*-pyran (6e).

Prepared from 25.0 g of **7e** using **Procedure A** in 39% yield or from 34.0 g of **7e** in 33% yield using **Procedure B** and obtained as a colorless oil: bp 135–140 °C (0.05 mmHg); ^{1}H NMR (400 MHz, $CDCl_3$) δ 2.26 (t, *J* = 5.1 Hz, 2H), 2.36 (t, *J* = 5.0 Hz, 2H), 3.34 (d, *J* = 7.5 Hz, 2H), 3.69 (q, *J* = 5.2 Hz, 2H), 3.71 (q, *J* = 5.3 Hz, 2H), 5.35 (t, *J* = 6.9 Hz, 1H), 7.10 (d, *J* = 8.6 Hz, 2H), 7.25 (d, *J* = 8.0 Hz, 2H); ^{13}C NMR (100 MHz, $CDCl_3$) δ 29.8, 32.5, 36.9, 68.7, 69.6, 121.2, 128.5 (2C), 129.6 (2C), 131.6, 135.8, 139.7. Anal. Calcd. for $C_{13}H_{15}ClO$: C, 70.11; H, 6.79. Found: C, 70.35; H, 6.79.

General Procedure for Preparation of Phosphonium Salts (7).

Procedure A. A solution of equimolar amounts of triphenylphosphine and alkyl bromide in toluene was heated under reflux for 24 h under an Ar atmosphere. Upon cooling to rt, the precipitate was removed by filtration and washed with toluene to give the phosphonium salt which was used without further purification.

Procedure B. A solution of equimolar amounts of triphenylphosphine and alkyl bromide was stirred at 100 °C overnight under an Ar atmosphere. A minimal amount of CH_3CN was added to prevent the mixture from becoming a glass as the reaction progressed. The resulting viscous gel was washed with hexane, and the gel heated in vacuo (100 °C, 2 mmHg) for 3 h to remove volatiles. The crude phosphonium salt was dissolved in CH_2Cl_2 and passed through a short plug of silica gel (eluent: CH_3OH/CH_2Cl_2, 1:19) to give the phosphonium salt as a white tacky solid. The tacky, hygroscopic phosphonium salt was further dried in vacuo (100 °C, 2 mmHg) and used without further purification.

Procedure C. Following the literature procedure [9], a solution of alkyl bromide (50 mmol) and triphenylphosphine (55 mmol) in benzene (100 mL) was heated under reflux for 48 h under an Ar atmosphere. The reaction was cooled to rt and the upper layer of the biphasic system decanted to remove excess triphenylphosphine and unreacted alkyl bromide. Additional benzene (100 mL) was added and heating under reflux continued for 30 min, and then the decantation process was repeated twice. The phosphonium salt was further dried in vacuo (130 °C, 1 mmHg) for several hours and used without further purification.

Propyltriphenylphosphonium Iodide (7a).

Prepared according to **Procedure A** in 90% yield and obtained as a colorless solid: mp 220–222 °C (dec); ^{1}H NMR (400 MHz, $CDCl_3$) δ 1.25 (td, J_1 = 7.2 Hz, J_2 = 1.4 Hz, 3H), 1.61–1.75 (m, 2H), 3.65–3.72 (m, 2H), 7.67–7.74 (m, 6H), 7.77–7.86 (m, 9H); ^{13}C NMR (100 MHz, $CDCl_3$) δ 15.4 (d, J_{PC} = 17 Hz), 16.5 (d, J_{PC} = 4 Hz), 24.8 (d, J_{PC} = 50 Hz), 118.1 (d, J_{PC} = 85 Hz, 3C), 130.5 (d, J_{PC} = 12 Hz, 6C), 133.7 (d, J_{PC} = 10 Hz, 6C), 135.1 (d, J_{PC} = 3 Hz, 3C); ^{31}P {^{1}H} NMR (162 MHz, $CDCl_3$) δ 25.3.

Pentyltriphenylphosphonium Iodide (7b).

Prepared according to **Procedure A** in 88% yield and obtained as a colorless solid: mp 166–168 °C; ^{1}H NMR (400 MHz, $CDCl_3$) δ 0.82 (t, J = 7.3 Hz, 3H), 1.28–1.33 (m, 2H), 1.62–1.64 (m, 2H), 2.16 (s, 2H), 3.64–3.70 (m, 2H), 7.67–7.74 (m, 6H), 7.77–7.86 (m, 9H); ^{13}C NMR (100 MHz, $CDCl_3$) δ 13.6, 22.2, 22.3 (d, J_{PC} = 5 Hz), 23.1 (d, J_{PC} = 50 Hz), 32.4 (d, J_{PC} = 16 Hz), 118.2 (d, J_{PC} = 85 Hz, 3C), 130.5 (d, J_{PC} = 13 Hz, 6C), 133.7 (d, J_{PC} = 10 Hz, 6C), 135.1 (d, J_{PC} = 3 Hz, 3C); ^{31}P {^{1}H} NMR (162 MHz, $CDCl_3$) δ 25.6.

2-(*trans*-4'-Pentylcyclohexyl)ethyltriphenylphosphonium Bromide (7c).

A solution of triphenylphosphine (6.71 g, 25.6 mmol) and 1-bromo-2-(*trans*-4'-pentylcyclohexyl)ethane (**10**, 5.00 g, 19.2 mmol) was stirred at 100 °C overnight under an Ar atmosphere. A minimal amount of CH_3CN was added to prevent the mixture from becoming a glass as the reaction progressed. The resulting viscous gel was washed with hexane, and the gel heated in vacuo (100 °C, 2 mmHg) for 3 h to remove volatiles. The

crude phosphonium salt (11.71 g) was dissolved in CH_2Cl_2 and passed through a short plug of silica gel (eluent: CH_3OH/CH_2Cl_2, 1:19) to give the phosphonium salt **7c** as a white, tacky, hygroscopic solid. The phosphonium salt was further dried in vacuo (100 °C, 2 mmHg) to give 8.05 g of **7c** (80% yield) as a colorless solid which was used without further purification: mp 183–184 °C; ^{1}H NMR (400 MHz, $CDCl_3$) δ 0.84 (t, J = 6.9 Hz, 3H), 0.76–0.93 (m, 4H), 1.05–1.14 (m, 3H), 1.15–1.30 (m, 6H), 1.42–1.56 (m, 3H), 1.65–1.73 (m, 2H), 1.77–1.86 (m, 2H), 3.66–3.76 (m, 2H), 7.67–7.74 (m, 6H), 7.77–7.86 (m, 9H); ^{13}C NMR (100 MHz, $CDCl_3$) δ 14.0, 20.8 (d, J_{PC} = 55 Hz), 22.6, 26.5, 29.6, 32.1, 32.6 (2C), 32.7 (2C), 37.1, 37.5, 38.1, 118.32 (d, J_{PC} = 86 Hz, 3C), 130.4 (d, J_{PC} = 13 Hz, 6C), 133.7 (d, J_{PC} = 10 Hz, 6C), 134.9 (3C); ^{31}P {^{1}H} NMR (162 MHz, $CDCl_3$) δ 26.4. Anal. Calcd. for $C_{31}H_{40}BrP$: C, 71.12; H, 7.70. Found: C, 71.40; H, 7.77.

4-Chlorophenethyltriphenylphosphonium Bromide (7e) [10].

Prepared according to **Procedure B** (65% yield) or **Procedure C** (86% yield) and obtained as light yellow glass. ^{1}H NMR data was consistent with the literature values [10].

Preparation of 4-Chlorophenethyl Bromide (8) [11].

In a 1 L, 3-necked flask, a solution of methanol (300 mL), H_2SO_4 (30 mL), and 4-chlorophenylacetic acid (30 g, 176 mmol) was heated under reflux for 12 h. The reaction was cooled to rt, and excess CH_3OH removed in vacuo. H_2O (300 mL) was added, and the reaction mixture extracted with Et_2O (4 × 75 mL). The organic layers were combined, washed with sat. $NaHCO_3$ solution (3 × 100 mL), dried ($MgSO_4$), filtered and evaporated

to afford 32.1 g (99% yield) of methyl 4-chlorophenylacetate as a slight yellow oil which was used with further purification: ^{1}H NMR (400 MHz, $CDCl_3$) δ 3.60 (s, 2H), 3.70 (s, 3H), 7.21 (d, *J* = 8.4 Hz, 2H), 7.30 (d, *J* = 8.4 Hz, 2H).

In a 1 L, 3 necked flask, a solution of methyl 4-chlorophenylacetate (32.1 g, 174 mmol) in anhydrous Et_2O (100 mL) was added dropwise to a suspension of $LiAlH_4$ (3.1 g, 81 mmol) in anhydrous Et_2O (200 mL) at 0 °C under an Ar atmosphere. The reaction was warmed to rt and gently heated under reflux overnight. The reaction was cooled to 0 °C and carefully quenched with H_2O (25 mL) and then 2 M KOH (50 mL). After 30 minutes, a white precipitation was removed by filtration and washed with Et_2O (100 mL). The filtrate was dried ($MgSO_4$), filtered and evaporated to leave 24.0 g (88% yield) of 4-chlorophenethyl alcohol as a colorless oil which was used without further purification: ^{1}H NMR (300 MHz, $CDCl_3$) δ 1.44 (br s, 1H), 2.88 (t, *J* = 6.6 Hz, 2H), 3.88 (br s, 2H), 7.16 (d, *J* = 8.5 Hz, 2H), 7.28 (d, *J* = 8.4 Hz, 2H); ^{13}C NMR (100 MHz, $CDCl_3$) δ 38.5, 63.4, 128.7 (2C), 130.3 (2C), 132.3, 137.0.

In a 1 L, 3-necked flask, a solution of 4-chlorophenethyl alcohol (23.0 g, 147 mmol) in anhydrous toluene (100 mL) under an Ar atmosphere was carefully treated dropwise with PBr_3 (6 mL, 64 mmol) at 0 °C. The solution was warmed to rt and heated under reflux for 3 h. The resulting orange suspension was re-cooled to 0 °C and treated with a 1:1 $NaHCO_3/Na_2S_2O_3$ solution (by mass, 100 mL). The biphasic system was filtered to remove insoluble material, and the aqueous layer removed. The organic layer was further washed with the 1:1 solution of $NaHCO_3/Na_2S_2O_3$ (2 × 100 mL), dried ($MgSO_4$), filtered and evaporated. The resulting light yellow oil was passed through a short plug of silica gel (eluent: hexanes) and further purified by bulb-to-bulb distillation (60 °C, 0.3 mmHg)

to give 28.6 g (90% yield) of 4-chlorophenethyl bromide (**8**) as a colorless oil: ^{1}H NMR (400 MHz, $CDCl_3$) δ 3.13 (t, *J* = 7.4 Hz, 2H), 3.54 (t, *J* = 7.4 Hz, 2H), 7.15 (d, *J* = 8.5 Hz, 2H), 7.29 (d, *J* = 8.4 Hz, 2H); ^{13}C NMR (100 MHz, $CDCl_3$) δ 32.6, 38.5, 128.7 (2C), 130.0 (2C), 132.8, 137.2.

1-Bromo-2-(*trans*-4-pentylcyclohexyl)ethane (10) [12,13].

A solution of 2-(*trans*-4-pentylcyclohexyl)acetaldehyde (**9**, 10.8 g, 55 mmol) [14] in CH_3OH (20 mL) was added dropwise to a suspension of $NaBH_4$ (3.00 g, 79 mmol) in CH_3OH (100 mL). The reaction was stirred until the vigorous effervescence had ceased, and the reaction progress was monitored by ^{1}H NMR. Excess $NaBH_4$ was quenched carefully by the addition of H_2O (100 mL), insoluble material removed by filtration and excess CH_3OH removed in vacuo. The aqueous layer was acidified with 10% HCl (100 mL) and the solution extracted with CH_2Cl_2 (3 × 30 mL). The organic layers were combined, dried ($MgSO_4$), filtered and evaporated to give a yellow oil. The oil was passed through a short plug of silica gel (eluent: CH_2Cl_2) and further purified by bulb-to-bulb distillation (145 °C, 0.15 mmHg) to afford 5.9 g (54% yield) of 2-(*trans*-4-pentylcyclohexyl)ethanol as a colorless oil: R_f = 0.2 (CH_2Cl_2); ^{1}H NMR (400 MHz, $CDCl_3$) δ 0.88 (t, *J* = 7.0 Hz, 3H), 0.82–0.98 (m, 3H), 1.10–1.37 (m, 10H), 1.46 (q, *J* = 6.8 Hz, 2H), 1.73 (br d, *J* = 9.4 Hz, 2H), 1.68–1.80 (m, 2H), 3.44 (s, 1H), 3.68 (q, *J* = 6.2 Hz, 2H); ^{13}C NMR (100 MHz, $CDCl_3$) δ 14.1, 22.7, 26.6, 32.2, 33.2 (2C), 33.3 (2C), 34.4, 37.4, 37.7, 40.4, 61.0.

In a 500 mL, 3 necked flask, a solution of 2-(*trans*-4-pentylcyclohexyl)ethanol (5.9 g, 30 mmol) in anhydrous toluene (75 mL) under an Ar atmosphere was carefully treated

dropwise with PBr_3 (1.4 mL, 15 mmol) at 0 °C. The solution was warmed to rt and heated under reflux for 3 h. The resulting orange suspension was re-cooled to 0 °C and treated with a 1:1 $NaHCO_3/Na_2S_2O_3$ solution (by mass, 75 mL). The biphasic system was filtered to remove insoluble material and the aqueous layer removed. The organic layer was further washed with the 1:1 solution of $NaHCO_3/Na_2S_2O_3$ (2 × 75 mL). The organic layer was dried ($MgSO_4$), filtered and evaporated. The resulting light yellow oil was passed through a short plug of silica gel (eluent: hexanes) and further purified by bulb-to-bulb distillation (140 °C, 0.15 mmHg) to give 6.1 g (78% yield) of 1-bromo-2-(*trans*-4-pentylcyclohexyl)ethane (**10**) as a colorless oil: ^{1}H NMR (400 MHz, $CDCl_3$) δ 0.88 (t, *J* = 7.1 Hz, 3H), 0.83–0.98 (m, 4H), 1.10–1.19 (m, 3H), 1.20–1.33 (m, 7H), 1.35–1.45 (m, 1H), 1.69–1.79 (m, 6H), 3.44 (t, *J* = 7.2 Hz, 2H); ^{13}C NMR (100 MHz, $CDCl_3$) δ 14.1, 22.7, 26.6, 32.0, 32.2, 32.6 (2C), 33.0 (2C), 36.5, 37.3, 37.7, 40.4. Anal. Calcd. for $C_{13}H_{25}Br$: C, 59.77; H, 9.65. Found: C, 59.92; H, 9.68.

Preparation of [*closo*-1-CB_9H_8-1-$SC_{19}H_{42}$-10-I] (11c).

Compound **11c** was obtained in 35% yield (after recrystallization: *iso*-octane/toluene followed by CH_3CN) from [*closo*-1-CB_9H_8-1-$SCHNMe_2$-10-I] (**12**, 0.316 g, 0.95 mmol) and dibromide **1c** (0.360 g, 0.95 mmol), according to the procedure described for **11d** (Cs_2CO_3/Bu_4NBr) or in 25% yield (after recrystallization) from **12** (0.263 g, 0.79 mmol) and dibromide **1c** (0.323 g, 0.79 mmol) using the published procedure [15] for **11b** ($NMe_4^+OH^-\cdot 5H_2O$, 0.286 g, 1.58 mmol): mp 217 °C (DSC); ^{1}H NMR (400 MHz, $CDCl_3$) δ 0.80–2.80 (m, 8H), 0.88 (t, *J* = 7.0 Hz, 3H), 0.81–0.97 (m, 4H), 1.10–1.20 (m, 4H), 1.21–1.34 (m, 7H), 1.40–1.49 (m, 2H), 1.61–1.88 (m, 8H), 2.54 (br d, *J* = 13.8 Hz, 2H),

3.67 (t, *J* = 12.7 Hz, 2H), 4.09 (br d, *J* = 11.9 Hz, 2H); ^{11}B NMR (128 MHz, $CDCl_3$) δ −19.5 (d, *J* = 146 Hz, 4B), −14.6 (d, *J* = 143 Hz, 4B), 24.9 (s, 1B). Anal. Calcd for $C_{19}H_{42}B_9IS$: C, 43.32; H, 8.04. Found: C, 43.42; H, 8.08.

Preparation of [*closo*-1-CB_9H_8-1-$SC_{17}H_{32}$-10-I] (11d).

A suspension of [*closo*-1-CB_9H_8-1-$SCHNMe_2$-10-I] (**12**, 2.80 g, 8.40 mmol), dibromide **1d** (3.20 g, 8.52 mmol), Cs_2CO_3 (8.30 g, 25.5 mmol) and $Bu_4N^+Br^-$ (0.50 g) in CH_3CN (250 mL) was stirred and gently heated under reflux overnight. The suspension was filtered through Celite, the filtrate evaporated, the resulting oily residue dissolved in CH_2Cl_2 and the solution washed with 5% HCl. The organic layer was separated, dried (Na_2SO_4), solvent evaporated, and the semi-solid residue passed through a plug of silica gel (eluent: CH_2Cl_2/hexane, 2:1). Solvents were evaporated, and the solid residue was washed with hot hexane to give 2.29 g of a white crystalline solid. Recrystallization from *iso*-octane/toluene mixture followed by CH_3CN gave 1.49 g (36% yield) of analytically pure sulfonium derivative **11d**: mp 241 °C (DSC); ^{1}H NMR (500 MHz, $CDCl_3$) δ 0.80–2.80 (m, 8H), 0.94 (t, *J* = 7.3 Hz, 3H), 1.64 (sext, *J* = 7.3 Hz, 2H), 1.70–1.80 (m, 3H), 1.86 (br q, *J* = 13.1 Hz, 2H), 2.53–2.60 (m, 4H), 2.70 (t, *J* = 7.3 Hz, 2H), 3.66 (br t, *J* = 12.8 Hz, 2H), 4.10 (br d, *J* = 12.3 Hz, 2H), 7.09 (d, *J* = 7.7 Hz, 2H), 7.14 (d, *J* = 7.7 Hz, 2H); ^{11}B NMR (128 MHz, CD_2Cl_2) δ −19.7 (d, *J* = 138 Hz, 4B), −14.8 (d, *J* = 154 Hz, 4B), 24.6 (s, 1B). Anal. Calcd for $C_{17}H_{32}B_9IS$: C, 41.44; H, 6.55. Found: C, 41.54; H, 6.63.

2. References

1. Krasovskiy, A.; Knochel, P. *Synthesis* **2006,** 890–891. doi:10.1055/s-2006-926345
2. Landini, D.; Montanari, F.; Rolla, F. *Synthesis* **1978,** 771–773. doi:10.1055/s-1978-24888
3. Thomas, J.; Clough, D. *J. Pharm. Pharmacol.* **1963,** *15,* 167–177.
4. Irwin, A. J.; Lok, K. P.; Huang, K. W.-C.; Jones, J. B. *J. Chem. Soc., Perkin Trans. 1* **1978,** 1636–1642.
5. Wu, G.; Huang, M.; Richards, M.; Poirier, M.; Wen, X.; Draper, R. W. *Synthesis* **2003,** 1657–1660. doi:10.1055/s-2003-40878
6. Organ, M. G.; Avola, S.; Dubovyk, I.; Hadei, N.; Kantchev, E. A. B.; O'Brien, C. J.; Valente, C. *Chem.–Eur. J.* **2006,** *12,* 4749–4755. doi:10.1002/chem.200600206
7. Day, J. N. E.; Thorpe, J. F. *J. Chem. Soc., Trans.* **1920,** *117,* 1465–1474.
8. Leotta, G. J., III; Overman, L. E.; Welmaker, G. S. *J. Org. Chem.* **1994,** *59,* 1946. doi:10.1021/jo00086a065
9. Nicolaou, K. C.; Yue, E. W.; la Greca, S.; Nadin, A.; Yang, Z.; Leresche, J. E.; Tsuri, T.; Naniwa, Y.; de Riccardis, F. *Chem.–Eur. J.* **1995,** *1,* 467–494. doi:10.1002/chem.19950010712
10. Okuma, K.; Yoshitake, K.; Izaki, T.; Yoshida, K.; Shioji, K. *Bull. Chem. Soc. Jpn.* **2007,** *80,* 1785–1790. doi:10.1246/bcsj.80.1785
11. Lambert, J. B.; Mark, H. W.; Magyar, E. S. *J. Am. Chem. Soc.* **1977,** *99,* 3059–3067. doi:10.1021/ja00451a035

12. Dąbrowski, R.; Dziaduszek, J.; Szczuciński, T.; Parka, J. *Mol. Cryst. Liq. Cryst.* **1995,** *260*, 201–215. doi:10.1080/10587259508038692

13. Fearon, J. E.; Gray, G. W.; Ifill, A. D.; Toyne, K. J. *Mol. Cryst. Liq. Cryst.* **1985,** *124*, 89–103. doi:10.1080/00268948508079467

14. Jankowiak, A.; Januszko, A.; Ringstrand, B.; Kaszynski, P. *Liq. Cryst.* **2008,** *35*, 65–77. doi:10.1080/02678290701744561

15. Ringstrand, B.; Kaszynski, P.; Januszko, A.; Young, V. G., Jr. *J. Mater. Chem.* **2009,** *19*, 9204–9212. doi:10.1039/b913701g
